# Acute High-Intensity Interval Exercise Improves Inhibitory Control Among Young Adult Males With Obesity

**DOI:** 10.3389/fpsyg.2020.01291

**Published:** 2020-06-25

**Authors:** Chun Xie, Brandon L. Alderman, Fanying Meng, Jingyi Ai, Yu-Kai Chang, Anmin Li

**Affiliations:** ^1^School of Psychology, Shanghai University of Sport, Shanghai, China; ^2^Department of Kinesiology and Health, Center of Alcohol and Substance Use Studies, Rutgers, The State University of New Jersey, New Brunswick, NJ, United States; ^3^Institute of Physical Education, Huzhou University, Huzhou, China; ^4^Department of Physical Education, National Taiwan Normal University, Taipei, Taiwan; ^5^Institute for Research Excellence in Learning Science, National Taiwan Normal University, Taipei, Taiwan

**Keywords:** HIIE, executive function, inhibitory control, obesity, P3, LPP

## Abstract

**Objective:**

The aim of the present study was to examine the influence of acute high-intensity interval exercise (HIIE) on neural and behavioral measures of inhibitory control in young male adults with obesity.

**Design:**

The present study employed a within-subjects design.

**Methods:**

Sixteen male adults with obesity [body mass index (BMI) > 28 kg/m^2^] were recruited. Reaction time and response accuracy of the Flanker task as well as P3 and late positive potential (LPP) components of the event-related potential (ERP) were measured following HIIE and a sedentary control, in counterbalanced order. The HIIE session consisted of 30 min of stationary cycle exercise (5-min warm-up, 20-min HIIE, and a 5-min cool-down), whereas the control condition consisted of a time and attention-matched sedentary resting session.

**Results:**

Faster response times were observed following HIIE regardless of Flanker task condition. Faster and more accurate responses were also observed for congruent relative to incongruent conditions across both sessions. Relative to the neuroelectric data, acute HIIE resulted in increased LPP amplitude but did not affect P3 amplitude.

**Conclusion:**

Collectively, a single bout of HIIE has a general beneficial effect on basic information processing and inhibitory control among young adult males with obesity. Acute HIIE was found to impact LPP amplitude, but not the P3, which may suggest a modulation in the ability to successfully maintain attention and filter irrelevant information to achieve successful cognitive inhibition. Future research is warranted to extend these findings to a larger sample size that includes both genders, other cognitive functions, and a comparison of different modes of exercise.

## Introduction

Obesity is a global public health concern that is associated with an increased risk of adverse health conditions including cardiovascular disease and diabetes (Ng et al., 2014) as well as impaired cognition, including executive function, attention, decision making, and memory (Smith et al., 2011; Prickett et al., 2015; Yang et al., 2018). Obesity has also been associated with impairments in inhibitory control. Inhibitory control, a core domain of the executive function, reflects as the ability to suppress inappropriate actions or override the processing of task-irrelevant or distracting information (Bari and Robbins, 2013). Inhibitory control is supported and regulated by multiple bidirectional neural connections, and has been shown to play a crucial role in the ability to inhibit excessive eating behaviors to maintain a healthy weight (Hwang et al., 2010). For instance, individuals with obesity performed worse (slower and less accurately) across several inhibitory control tasks (e.g., Flanker and Stroop inhibitory control tasks), suggesting an impaired ability to override interference information among individuals with obesity relative to their normal-weight counterparts (Kamijo et al., 2012; Gameiro et al., 2017). A recent meta-analysis also reported a moderate inverse effect size for inhibitory control deficits among overweight and obese individuals (Yang et al., 2018). Atrophy of prefrontal cortical regions implicated in cognitive control has been found in individuals with obesity, providing evidence of obesity-impaired cognitive inhibition through both structural and functional brain imaging data (García-García et al., 2015).

Robust and consistent evidence has shown that acute exercise that represents a single bout of exercise elicits beneficial effects on cognitive function (Etnier et al., 1997; Ferris et al., 2007; Chang et al., 2012b; Etnier and Chang, 2019; Wilke et al., 2019) and the beneficial effects from acute exercise have also extended to inhibitory cognitive control (Themanson and Hillman, 2006; Drollette et al., 2014; Chu et al., 2015; Hsieh et al., 2018). However, studies that have investigated acute exercise and inhibitory control have almost exclusively relied on traditional aerobic or resistance modes of exercise (Harveson et al., 2016; Hsieh et al., 2016; Ludyga et al., 2017) and the acute effect of other modes of exercise, particularly those that are engaged in by an increasing number of adults, require consideration to advance our knowledge regarding both efficacy and mechanisms of action (Chang et al., 2019).

High-Intensity Interval Training (HIIT) is a popular and emerging exercise mode that has been shown to elicit beneficial health effects in an efficient manner compared to more long-duration continuous exercise. HIIT generally involves repeated bouts of short to moderate duration high-intensity interval exercise (HIIE) with a larger than anaerobic threshold intensity and interspersed with recovery periods or light exercise (Laursen and Jenkins, 2002; Weston et al., 2014). The HIIE has been linked to improved physical fitness including cardiorespiratory fitness (O’Donovan et al., 2005), skeletal muscle metabolism (Rognmo et al., 2004), and body composition in adults with obesity (Wewege et al., 2017). HIIE has also been shown to positively impact cognitive function. For example, adults exhibited faster overall reaction times and improved accuracy in the Flanker task following 9 min of HIIE compared to a control condition (Kao et al., 2017) and adult males have demonstrated an improvement in inhibitory control (i.e., Stroop test performance) during the post-exercise recovery period after a 28 min bout of cycling HIIE (Tsukamoto et al., 2016). Yet, these studies have only been targets on healthy adults with normal weight, and whether the positive effects of HIIE on cognitive function extend to an adult population with obesity remains unclear. Though only a few studies have examined the effects of exercise on cognition in obese populations, Russo et al. (2017) found that aerobic exercise may improve cognition in obese adults. Previous studies have also demonstrated that HIIE may be more effective than continuous aerobic exercise at improving cognition among normal-weight adults (Tsukamoto et al., 2016; Kao et al., 2017, 2018); therefore, it is possible that HIIE may be an effective mode of exercise for improving cognition in individuals with obesity. One previous study was conducted to examine the impact of HIIE on cognition in obese adults, and showed an improvement in cognitive function, particularly in inhibitory control (Stroop test) after HIIE (Drigny et al., 2014). However, this study was a long-term intervention with HIIE that included only six participants and lacked a control group. Additionally, Quintero et al. (2018) conducted an acute HIIE study on cognitive function in overweight male adults and found positive effects on cognitive inhibition. However, the sample size of their study was relatively small (six participants in HIIE group) and the population was overweight rather than obese. Therefore, the effects of acute HIIE on cognitive function among obese adults is still preliminary, and further investigation is warranted.

Event-related brain potentials (ERPs) from the ongoing electroencephalographam (EEG) represents a sensitive, high temporal resolution of underlying neurocognitive mechanisms between the presentation of a stimulus to beyond response execution processes, and have been commonly utilized to examine the relation between acute exercise and inhibition aspect of executive function (Kamijo et al., 2007; Chu et al., 2015; Wang et al., 2015; Kao et al., 2017). Notably, the majority of studies to date have focused on the P3 (P300 or P3b) component of the ERP, and the acute exercise effect on other components (e.g., late positive potential, LPP) require further examination. The LPP is an ERP component with a more extended latency, which reflects high-order attention processes (Schupp et al., 2003; Carbine et al., 2018) and the influence of cognitive conflict on the later phase of processing (Ligeza and Wyczesany, 2017). Increased LPP amplitude to a stimulus is associated with the increased conscious allocation of attention (Schupp et al., 2003; Carbine et al., 2018) and cognitive control (Watson and Garvey, 2013; Carbine et al., 2018), reflecting a linkage between LPP and inhibitory control.

There has been limited research conducted to examine the influence of HIIE on cognitive function among normal weight adults (Tsukamoto et al., 2016; Kao et al., 2017, 2018) or among overweight or obese adults (Drigny et al., 2014; Quintero et al., 2018). Therefore, it remains important to investigate the effects of acute HIIE on cognitive function among obese adults. It is also unknown how HIIE may affect cognitive function among obese individuals – that is, the precise underlying mechanisms involved. Therefore, the aim of current study was to examine the effects of acute HIIE on cognitive function in young adults with obesity. Specifically, the inhibitory control domain of executive function was assessed via both behavioral and electrophysiological measures using the P3 and LPP components following HIIE. It was hypothesized that an acute HIIE session would facilitate inhibitory control performance among young adults with obesity, and this improvement would be demonstrated by increased P3 and LPP component amplitudes.

## Materials and Methods

### Participants

Sixteen male adults between the age of 18 and 35 years were recruited from the Fengxian district of Shanghai. Eligible participants were screened by the following requirements: (a) obesity status: body mass index (BMI) > 28 kg/m^2^ (the value represents the cutoff point obesity on the Asia weight categories); (b) right-handed dominance, (c) no history or presence of endocrine and cardiovascular diseases; (d) no history or presence of neurological disorders or head injury with loss of consciousness; (e) no current use of medication that could affect blood glucose and insulin levels, or any weight loss supplement; (f) “No” answer in any questions of Physical Activity Readiness Questionnaire; and (g) normal or corrected-to-normal vision. All participants provided written informed consent in accordance with the Institutional Review Board at the Shanghai University of Sport (#102772019RT005). The data of the demographic characteristics are provided in Table 1.

**TABLE 1:** Demographic characteristics of study participants (means ± SD).

Age (years)	24.50 ± 5.09
Height (m)	1.77 ± 0.06
Weight (kg)	108.11 ± 16.72
BMI (kg/m^2^)	34.34 ± 4.39
Digital span	
Forward	14.44 ± 2.28
Backward	9.69 ± 3.63
Education level	
High school	2
College	11
Post-Graduate	2
VO_2peak_ (ml/kg/min)	29.74 ± 12.38
Basal metabolic rate (kJ/m^2^/h)	2072.29 ± 191.84
DEBQ	
Restrained eating	3.07 ± 0.37
External eating	3.37 ± 0.62
Emotional eating	2.16 ± 0.74

*BMI, body mass index; DEBQ, Dutch Eating Behavior Questionnaire.*

### Cardiovascular Fitness Assessment

The YMCA submaximal ergometer exercise test (MONARK 894E, Sweden) was conducted to assess participants’ cardiorespiratory fitness (i.e., maximum oxygen consumption, VO_2max_). The protocol comprises several consecutive 3-min stages. During the test, a Polar heart rate (HR) monitor was continuously worn to monitor HRs. In the first stage, participants were instructed to cycle at a cadence of 50 rpm at a workload of 25 W (150 kg/min, 0.5 kp). The workloads of the following stages were calibrated according to the HRs of the last 15–30 s (i.e., the steady-state HRs) of the first stage. For instance, the subsequent workloads of the second and third stages were set to 100 W (600 kg/min, 2 kp) and 125 W (750 kg/min, 2.5 kp), respectively, if participant’s HRs were lower than 86 bpm. If the steady-state HRs were between 86 and 100 bpm, the workloads of the second and third stages were set to 75 W (450 kg/min, 1.5 kp) and 100 W (600 kg/min, 2 kp), respectively. Lastly, if HR was higher than 100 bpm, the second and third stages were set to 50 W (300 kg/min, 1 kp) and 75 W (450 kg/min, 1.5 kp), respectively. If HR did not achieve steady state in each stage, an additional 1-min cycling was added. The original 6–20 rating of perceived exertion (RPE) (Borg, 1982) was documented every 2 min throughout the test.

### Flanker Task

The computerized Flanker task was utilized to assess several aspects of cognitive function (e.g., basic information processing and the inhibitory control aspect of the executive function) implemented on E-Prime software (version 2.0, Psychological Software Tools, Pittsburgh, PA, United States). Each stimulus was composed of five arrows (i.e., one central target and four surrounding flankers) and was presented on the center of the 27-inch LCD screen with a gray background at a distance of 90 cm from participants. The direction of the center target was either the same as the flankers (e.g., →→→→→ or ←←←←←; the congruent condition) or opposite to the flankers (e.g., →→←→→ or ←←→←←; the incongruent condition). A black cross was flashed for 500 ms as a cue to signal the beginning of the trial, followed by the presentation of stimuli for 1000 ms. Once a response was made, a blank intertrial interval with a various time interval (i.e., 600–800 ms) was presented. Participants were asked to put their right index fingers on the number “5” key but not press it, then press the key on the keyboard (number “4” key for left directions and number “6” key for the right directions of the central target, respectively) as fast and accurate as possible. After the key was pressed, the participants were instructed to place their right index fingers back to the “5” key to await the next stimuli. Incorrect responses, responses made beyond 1000 ms after the stimulus onset, or reaction time more than +3 standard deviations (SD) of each participant’s mean reaction time, were discarded from the further analysis. The task contained one practice block and two formal blocks of trials. In the practice block, there were 12 trials with an equal number of congruent and incongruent trial conditions. Participants were required to pass the practice block of trials with an accuracy rate higher than 80%. Each of the formal blocks contained 100 trials, and each of the four stimulus trial types occupied 1/4 of the whole block of trials. The reaction time of accepted response and accuracy for the congruent and incongruent conditions were recorded as the indices of behavioral performance.

### Electrophysiological Recording and Analysis

The activity of continuous EEG was recorded from a 64 Ag/AgCl cap with electrodes located at the standard International 10–20 positions using a 1000 Hz sampling rate (Brain Products GmbH, Munich, Germany) throughout the Flanker task. The reference electrode was placed over FCz, and the ground electrode was the AFz. The vertical electrooculogram (VEOG) and the horizontal electrooculogram (HEOG) were recorded through the electrodes placed below the left eye, and the electrodes at lateral-orbitally of the right eye, respectively. All electrodes impedances were kept below 10 kΩ.

All EEG offline processing was conducted using Brain Vision Analyzer 2. The offline EEG data were initially re-referenced according to the average of the left and right mastoids, and the ocular correction was performed (Gratton et al., 1983). Artifact rejection was based on the exclusion of all epochs that exceeded ±100 V. The baseline was corrected (−200 to 0 ms pre-stimulus onset). The continuous EEG data were epoched into 1700-ms time windows (from 200 ms before to 1500 ms after the stimulus onset) for correct trials and then filtered with an IIR filter (low cutoff: 0.1 Hz, high cutoff: 30 Hz, slope: 48 dB/oct). The mean number of analyzable segments of congruent and incongruent conditions were 94.41 ± 7.67 (range 70–100) and 90.84 ± 9.63 (range 60–100), respectively. P3 and LPP component amplitudes were quantified as the area-averaged amplitude within 300–400 ms and 600–1100 ms after the stimulus onset from the Fz electrode (Luijten et al., 2016; Kao et al., 2018), respectively, for both the congruent and incongruent conditions.

### Experimental Procedure

Participants were invited to the laboratory on two separate occasions, with the HIIE and control sessions performed in a randomized order across participants. The written informed consent, the physical activity readiness questionnaire (Thomas et al., 1992), and a demographics survey were completed in the participant’s first visit. Intelligence indexed by the Digit Span Forward and Backward tests from the Wechsler Adult Intelligence Scale-Third Edition (WAIS-III) (Wechsler, 1997) and socioeconomic status were also measured. Weight and height were measured using an electrical scale (Yaohua Weighing System Co., Shanghai, China) and a wall-mounted stadiometer (TANITA, Tokyo, Japan), respectively. The index of BMI was calculated using the standard formula as weight (kg)/height (m)^2^. Participants also completed the Dutch Eating Behavior Questionnaire (DEBQ), which is used to examine eating styles and contains 33 items to specifically explore restrained eating, external eating, and emotional eating (Van Strien et al., 1986). Previous research has demonstrated strong internal consistency of this measure in both genders and among both obese and normal-weight individuals (Cronbach’s alphas > 0.79) (Wardle, 1987).

**FIGURE 1:**
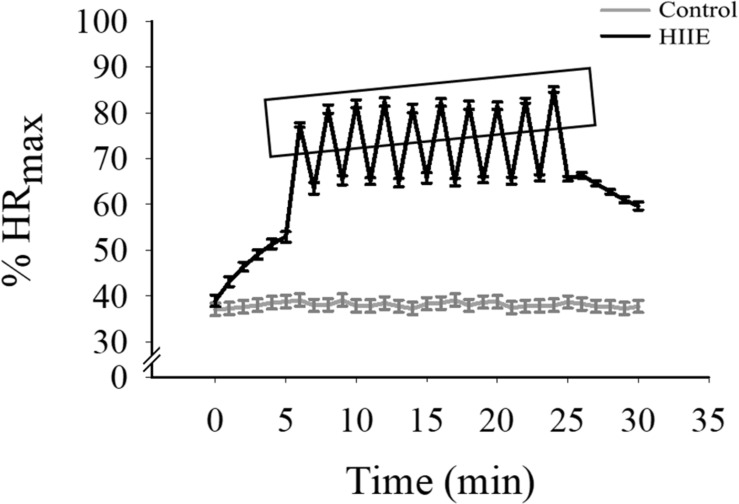
The alteration of the percentage of HR_max_ (%HR_max_) through both experimental conditions. The *x*-axis (1 min and 30 min) indicates %HR_max_ of the start and end of each condition, which is shown on the *y*-axis. The rectangle indicates %HR_max_ immediately following each HIIE interval.

For the experimental sessions (i.e., HIIE and control sessions), the HIIE session lasted approximately 30 min and involved 5 min of warm-up, a 10 × 1 min of 80–90% maximal HR (HR_max_) interspersed by 1 min of 50–65% HR_max_ active relax (for a total for 20 min), and 5 min of cool-down. The exercise was conducted via stationary cycle exercise because of biomechanical loads and knee safety concerns that may present among obese adults (Salih and Sutton, 2013). HRs and RPE were recorded every 1 min during the 20-min formal HIIE exercise bout. In the control session, participants sat silently with their eyes opened for 30 min, and HR was recorded throughout (Figure 1).

Within 15 min following the HIIE or control session, participants were seated comfortably in a chair while EEG was prepped. Then, the cognitive task was performed, and EEG was recorded throughout the task period. The cognitive task included three blocks, with one practice and two formal blocks. The total duration for each experimental session was roughly 50 min, and participants were debriefed following the final experimental session.

### Statistical Analysis

The study is a within-subject design, with session and stimuli conditions as within-subject factors. For the behavioral data, a 2 (session: HIIE vs. control) × 2 (congruency: congruent vs. incongruent) repeated-measures analysis of variance (ANOVA) was conducted for reaction time and response accuracy, respectively. For the ERP data, a 2 (session) × 2 (congruency) ANOVA was separately conducted for P3 and LPP component amplitudes at site Fz. Pairwise comparisons with Bonferroni adjustments were conducted as follow-up analyses. All statistical values were conducted with α = 0.05, where the Greenhouse–Geisser corrections and partial eta-squared ($\eta_{\text{p}}^{2}$) values are reported for significant statistics were observed.

## Results

### Behavioral Performance

**FIGURE 2:**
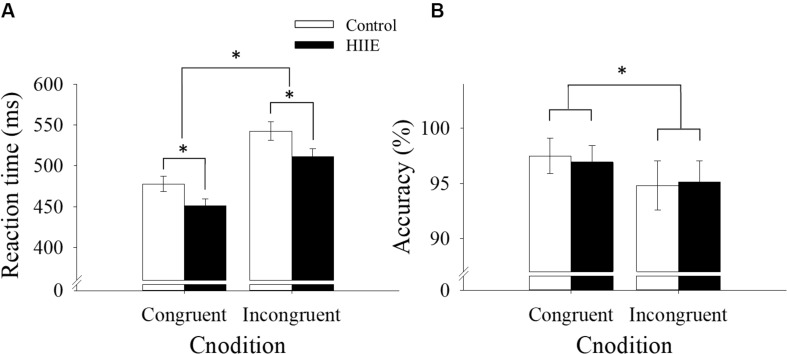
Behavioral reaction time **(A)** and accuracy data **(B)** for the Flanker task by session and congruency. ^∗^*p* < 0.05.

The ANOVA analysis of reaction time revealed a significant main effect of session [*F*(1,15) = 7.61, *p* < 0.05, $\eta_{\text{p}}^{2}$ = 0.34], with faster reaction time observed following the HIIE condition (481.28 ± 8.69 ms) compared with the control condition (510.29 ± 10.10 ms). The analysis also revealed a significant main effect of congruency [*F*(1,15) = 266.44, *p* < 0.001, $\eta_{\text{p}}^{2}$ = 0.95], with faster reaction time for congruent (464.69 ± 7.29 ms) compared with incongruent (526.87 ± 8.73 ms) trials. There was no significant interaction between session and congruency (*p* > 0.05). The behavioral reaction time data for the Flanker task are shown in Figure 2A.

The analysis of response accuracy revealed a significant main effect of congruency [*F*(1,15) = 14.64, *p* < 0.01, $\eta_{\text{p}}^{2}$ = 0.49], with more accurate responses observed for congruent (97.20 ± 1.5%) than incongruent (95.00 ± 2.00%) trials. However, no significant differences in response accuracy were observed between HIIE and the control session (*p* > 0.05), nor was there a significant interaction between session and congruency (*p* > 0.05). Response accuracy data are shown in Figure 2B.

### EEG Data

**FIGURE 3:**
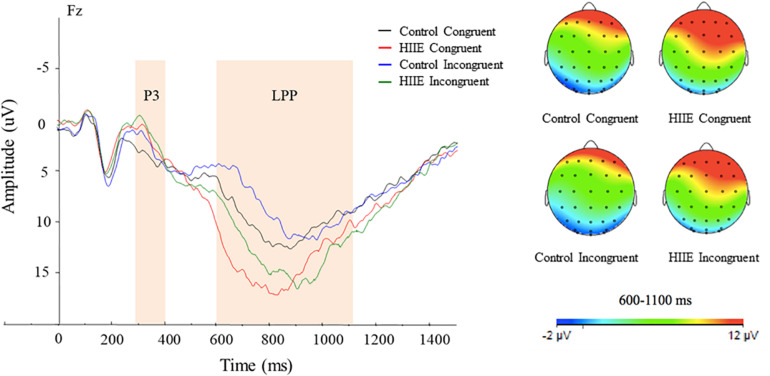
Grand-mean stimulus-locked ERP waveforms for each session and task condition. Stimulus-locked waveforms are shown for both congruency conditions at the midline electrode sites at Fz. Topographic maps of scalp neuroelectric activity for each session and task condition for LPP amplitude (right). The average area of P3 and LPP component amplitudes are shown in shaded regions. LPP component revealed larger amplitude observed following the HIIE (green and red) compared with the control condition (black and blue) at the Fz site. Topographic scalp distribution of the LPP revealed larger activation following the HIIE condition compared with the control condition at Fz regardless of congruency condition.

Figure 3 illustrates the mean stimulus-locked ERP waveform showing P3 and LPP component amplitudes for each session and stimulus condition.

#### P3 Component Amplitude

Analysis of P3 amplitude revealed no significant main effect for session or congruency (*p*s > 0.05). There was also no interaction between the experimental session and congruency for P3 amplitude (*p* > 0.05).

#### LPP Component Amplitude

Analysis of LPP component amplitude revealed a significant main effect of session [*F*(1,15) = 6.91, *p* < 0.05, $\eta_{\text{p}}^{2}$ = 0.32], with larger LPP amplitude observed following the HIIE (13.73 ± 1.86 μV) compared with the control condition (9.64 ± 1.43 μV) at Fz. There is also a significant main effect of congruency [*F*(1,15) = 6.69, *p* < 0.05, $\eta_{\text{p}}^{2}$ = 0.31], with larger LPP amplitude for congruent (12.27 ± 1.56 μV) compared with incongruent (11.10 ± 1.40 μV) task conditions. No significant interaction between session and congruency was found (*p*s > 0.05). In addition to the grand averaged ERPs, topographic scalp distribution of neuroelectric activity within the time frame of the LPP at site Fz has been shown in Figure 3.

## Discussion

The study examines the effect of an increasing popular mode of exercise, acute HIIE, on both behavioral and neural indices of inhibition control. Along with the few studies that have been conducted to determine the effects of a single bout of HIIE on inhibitory control, this is the first to examine the influence of this form of exercise on the P3 wave and LPP component among young adult males with obesity. The findings indicate that acute HIIE elicits faster reaction times, but no change in response accuracy, regardless of Flanker task congruency compared to a no-exercise control session. Additionally, while no acute exercise-related enhancements were observed for P3 amplitude, acute HIIE increased LPP component amplitude regardless of Flanker task conditions. Overall, these findings support previous research demonstrating that a single bout of moderate-intensity aerobic exercise increases inhibitory cognitive control (Pontifex et al., 2019) and extends these beneficial findings to HIIE and to young adult males with obesity.

Previous studies have shown that a single bout of HIIE improves inhibitory control performance among normal-weight adults (Alves et al., 2014; Tsukamoto et al., 2016; Kao et al., 2017, 2018) and the present study exhibited faster reaction time following acute HIIE that extends to male adults with obesity. The previous study also indicated decreased flanker task reaction time (Kamijo et al., 2007; Kao et al., 2017) and decreased Stroop reaction time following similar exercise protocols (Alves et al., 2014; Tsukamoto et al., 2016). Given that both of these two tasks are associated with the inhibitory domain of executive function or cognitive control (Harnishfeger and Bjorklund, 1993), the more efficient reaction times observed in these tasks suggest that acute HIIE enhances response speed (Kao et al., 2017) and improved efficiency in cognitive inhibition (Harnishfeger and Bjorklund, 1993; Kamijo et al., 2012), reflecting a facilitation in inhibitory control following acute HIIE. That this effect is observed in young males with obesity is also provocative since the exercise stimulus is likely to be more difficult relative to normal-weight counterparts performing similar overall workloads.

Behavioral performance of reaction time and accuracy was impaired in the more difficult incongruent relative to congruent task condition of the Flanker task, an effect that is consistently found in the literature (Alderman et al., 2016). These results have often been interpreted as a “conflict effect,” suggesting an increase in the task difficulty during incongruent task trials (Eriksen and Eriksen, 1974). In the incongruent task condition, a greater amount of interference control is involved, resulting in response delays, which is due to the fact that flanking stimuli provoked by the activation of the incorrect response compete with the correct response produced by the central target stimulus (Spencer and Coles, 1999; Larson et al., 2014). Notably, the improvement in cognition observed following the acute HIIE not only occurred for congruent but also for incongruent task conditions. Given that the congruent condition reflects relative basic information processing, whereas the incongruent condition reflects high-order inhibition, our results suggest that acute HIIE may have an overall general facilitating effect on cognitive function. These results are somewhat in contrast with studies that have demonstrated a selective improvement (e.g., faster cognitive processing speed) in the incongruent condition but not in congruent or neutral conditions after acute HIIE (Kamijo et al., 2007; Kao et al., 2017). Drollette et al. (2014) found that participants with lower inhibitory capacity may be among those who benefit the most from single bouts of exercise. Therefore, it is possible that the obese individuals from this study have a less flexible cognitive function, particularly within the domain of inhibitory control, relative to their normal-weight counterparts, and acute HIIE may generally enhance both their basic cognitive functioning and high-order cognitive functions such as inhibitory control. This speculation of individual differences in acute exercise–cognition relationship warrants future investigation, particularly given the rise of obesity in nearly all developed countries around the world.

In opposition to our hypothesis, we did not observe an effect of HIIE on P3 amplitude, suggesting that the allocation of attentional resources or cognitive processes that may be indexed by the P3 is not influenced following HIIE among young obese male adults. These results contrast with many previously published studies that have observed an increased P3 amplitude following the acute exercise (Drollette et al., 2014; Chu et al., 2015; Hsieh et al., 2018); yet, it should be noted that these increased P3 amplitudes following acute exercise have only been shown following continuous moderate-intensity aerobic exercise. Indeed, Kao et al. (2018) led a recent study to investigate the short-term neurocognitive effects of acute HIIE and did not find any significant change in P3 component amplitude compared to a sedentary control condition, findings similar to those shown in this study. Collectively, the findings from these two studies suggest that the type or mode of exercise might be a significant moderator of the acute exercise-related enhancement in cognitive function. In addition, the duration of exercise might also be a critical modulator of any acute exercise effects. Kao et al. (2017) found a decrease in P3 amplitude and latency following a 9-min session of HIIE compared to seated rest. They reasoned that such decreases in P3 amplitude following HIIE could be regarded as greater neural efficiency because of less recruitment of neural resources accompanied by improved inhibitory control (Voss et al., 2011; Malinowski, 2013; Kao et al., 2017). However, the results were in contrast with their study in 2018 (Kao et al., 2018), which found no change in P3 amplitude after a 16-min session of acute HIIE, and thus one consideration for the difference may have been due to the duration of the exercise. Given that the exercise duration used in this study was similar to that used in the Kao et al. (2018) study, we expect that exercise duration might have an important influence on P3 component amplitude, particularly when incorporating HIIE protocols. Specifically, HIIE with longer durations might lead to limited effects on P3 amplitude, and the extent to which this influences overall behavioral performance remains to be examined. Lastly, previous studies have mostly examined normal-weight individuals and very few studies to date have focused on the acute HIIE effects on behavioral and neural exponents of inhibitory control among obese adults; therefore, the results from this study relative to behavioral performance outcomes of the P3 may be specific to this population.

Our findings that HIIE improved LPP amplitude is novel for several reasons. LPP reflects higher-order attentional processes (Schupp et al., 2003; Carbine et al., 2018) and the influence of cognitive conflict on the late phase of processing (Ligeza and Wyczesany, 2017). In addition, although we did not use an emotional stimulus in this study, LPP amplitude has also been interpreted as a global inhibition of activity within the visual cortex, reflecting a more adaptive emotional processing (Brown et al., 2012). Our findings demonstrate that following acute HIIE, LPP amplitude increases, suggesting a modulation in the ability to successfully maintain attention and filter irrelevant information to achieve successful cognitive inhibition involved in the Flanker task.

The major strengths of the study include the investigations of the acute HIIE effects on multiple aspects of cognitive function as well as its neuroelectric response in obese individuals; however, potential limitations should be proposed. Our study only included obese male adults in order to avoid the potential moderating role of sex (males vs. females). Along with the relatively small sample size, these findings should not be broadly generalized and future studies employing larger sample size with a specified recruitment of both genders are recommended to further examine the influence of HIIE on select aspects of cognitive function. Additionally, while inhibitory control is a core component of executive function, other distinguishable components including shifting and updating (Miyake et al., 2000) as well as planning (Chang et al., 2012a) should be studied. These distinct components might be differentially influenced by various features of acute HIIE and should be examined to provide a more complete picture of acute HIIE-related modulations of executive function. Furthermore, previous studies have indicated the physiological response to HIIE and more traditional forms of aerobic exercise are different for normal-weight adults. However, the effects and brain mechanisms involved in the acute effects of HIIE on cognition in obese individuals were unclear and thus it is recommended for future studies to compare the impact of both HIIE and aerobic exercise among obese individuals. Lastly, further investigation is also warranted to explore the dose–response relationship between acute HIIE and cognitive function, in terms of intensity, duration, and volume, to advance more precise exercise prescription for cognition.

## Conclusion

Collectively, a single bout of HIIE has a general beneficial effect on basic information processing and inhibitory control among young adult males with obesity. Acute HIIE was shown to impact LPP component amplitude, but not P3 component amplitudes, which may suggest a modulation in the ability to successfully maintain attention and filter irrelevant information to achieve successful cognitive inhibition. Future study is suggested to extend the examination to a larger sample size that includes both genders, other cognitive functions, and a comparison of varied exercise modes.

## Data Availability Statement

The datasets generated for this study are available on request to the corresponding authors.

## Ethics Statement

The studies involving human participants were reviewed and approved by the Institutional Review Board at the Shanghai University of Sport (#102772019RT005). The patients/participants provided their written informed consent to participate in this study.

## Author Contributions

CX, Y-KC, and AL: conceptualization. CX, FM, and JA: methodology. BA, FM, and JA: formal analysis. CX, BA, Y-KC, and AL: investigation. All authors: writing – original draft preparation and review and editing.

## Conflict of Interest

The authors declare that the research was conducted in the absence of any commercial or financial relationships that could be construed as a potential conflict of interest.
